# Psychological Scars and Physical Consequences: Linking PTSD to Cardiovascular Health

**DOI:** 10.1007/s12265-026-10783-7

**Published:** 2026-05-26

**Authors:** Alexa Corker, Griffin Oh, Kristine Y. DeLeon-Pennell

**Affiliations:** 1 College of Graduate Studies, Medical University of South Carolina, 171 Ashley Ave, Charleston, SC 29425 USA; 2 Department of Medicine, Division of Cardiology, Medical University of South Carolina, 171 Ashley Ave, Charleston, SC 29425 USA; 3 Research Service, Ralph H. Johnson Veterans Affairs Medical Center, 109 Bee St, Charleston, SC 29401 USA

**Keywords:** PTSD, Heart disease, Inflammation, Sex difference

## Abstract

Post traumatic stress disorder (PTSD) is defined as a mental health condition that occurs after experiencing a traumatic event. Patients diagnosed with PTSD have a 30-45% increase in cardiovascular disease (CVD) risk or cardiac-specific mortality even after adjusting for depression, demographic, clinical (e.g., age, blood pressure, body mass index (BMI), and tobacco use), and psychosocial factors. With this, the lifetime prevalence of PTSD has a notable sex disparity: women have a prevalence of approximately 10%, compared to 4% in men. Mechanisms explaining the increased risk of CVD in PTSD patients is unclear, leaving this patient population vulnerable as well as placing a substantial economic burden on the United States healthcare system. In this review, we will discuss the effect of PTSD on cardiovascular physiology, with an emphasis on sex differences, mechanisms of inflammation, and impaired ANS responses. In addition, we will summarize current and experimental therapeutics for PTSD that may help mitigate the risk of CVD.

## Introduction

Clinicians, researchers, and patients have long recognized the link between psychological stress and physical health. Some have suggested that the increased risk of disease is due to the strain that stress places on the adaptive capacity of individuals [1]. Post-traumatic stress disorder (PTSD) is defined as a mental health condition that occurs after experiencing a traumatic event. Findings from the National Comorbidity Survey Replication implicated PTSD-associated physiological dysregulation in a range of conditions including cardiovascular, autoimmune, and musculoskeletal disorders [2]. Patients diagnosed with PTSD have a 30-45% increase in cardiovascular disease (CVD) risk or cardiac-specific mortality even after adjusting for depression, demographic, clinical (e.g., age, blood pressure, cholesterol, body mass index (BMI), and tobacco use), and psychosocial factors [3, 4].

Incidence of PTSD and CVD are increasing both nationally and globally with minimal advances in readily available treatments. While many potential mechanisms have been proposed, our understanding of PTSD-associated effects on the body is still unclear. Clinical observations have demonstrated an increased inflammatory response with PTSD diagnosis including increased circulation of pro-inflammatory cytokines and the expansion and hyperactivation of immune cell populations [5–7]. Studies have indicated that the uncontrolled elevation in the inflammatory response is likely due to an impaired autonomic nervous system (ANS). Current research focusing on autoimmune disorders are now implicating restoring the balance within the ANS as a possible therapeutic target [8]. These research findings may point to beneficial targets for treating PTSD patients in the future.

In this review, we will discuss the effect of PTSD on cardiovascular physiology, with an emphasis on sex differences, mechanisms of inflammation, and impaired ANS responses. In addition, we will summarize approved and experimental therapeutics for PTSD that may help mitigate the risk of CVD in patients.

## Pathophysiology of PTSD and CVD

**Fig. 1:**
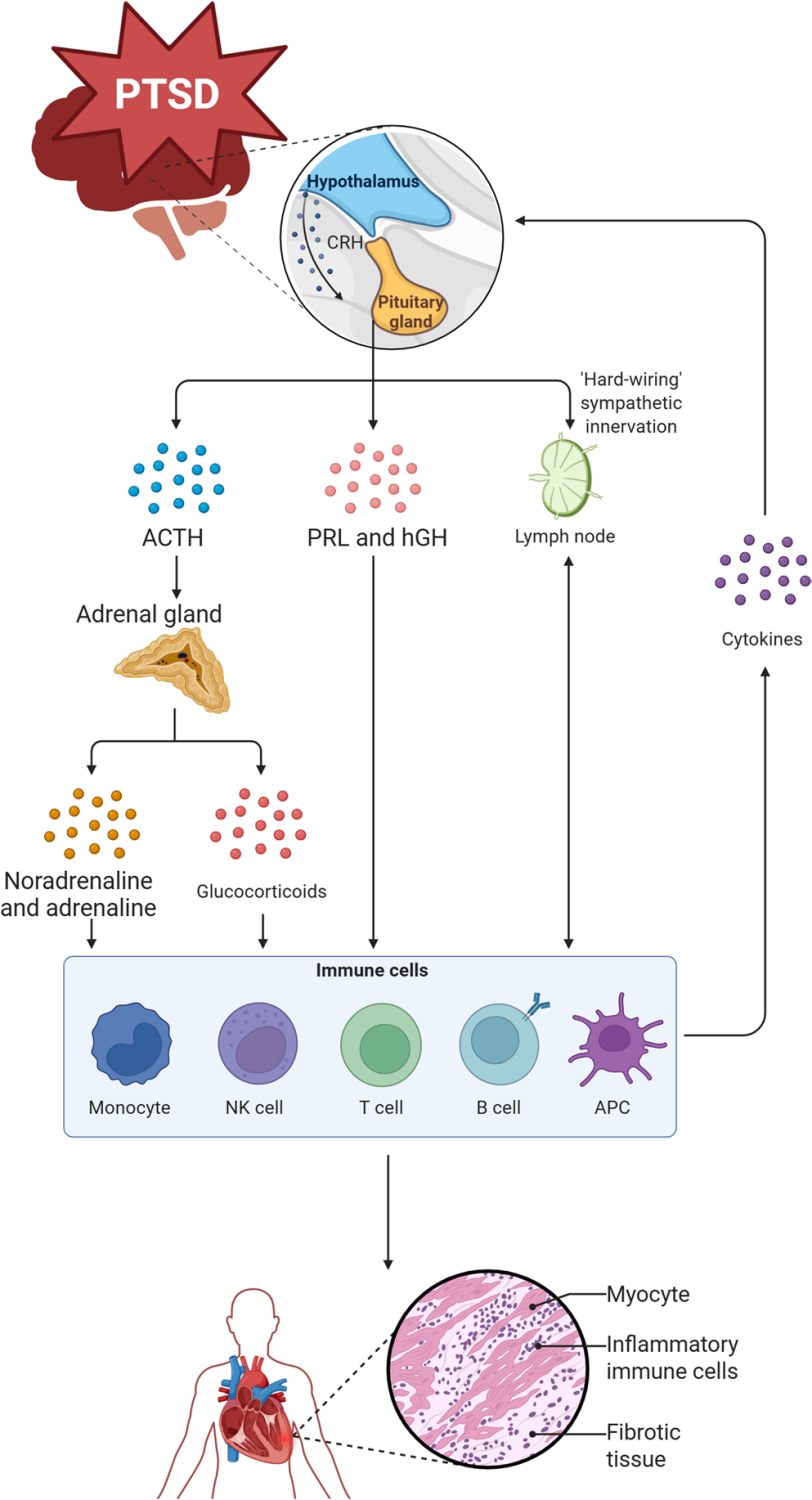
Proposed mechanisms linking PTSD to increased CVD risk. Post-traumatic stress disorder (PTSD) activates the hypothalamic-pituitary-adrenal (HPA) axis and sympathetic nervous system (SNS), leading to release of corticotropin-releasing hormone (CRH), adrenocorticotropic hormone (ACTH), prolactin (PRL), and human growth hormone (hGH). This cascade increases the secretion of glucocorticoids, noradrenaline, and adrenaline, which act on immune cells including monocytes and T cells. In parallel, sympathetic innervation of lymphoid tissues and stress-induced cytokine release amplify immune activation. Dysregulated neuroendocrine immune signaling promotes infiltration of inflammatory immune cells into cardiac tissue, driving myocyte injury and fibrosis, possibly contributing to the increased risk of cardiovascular disease in PTSD patients

After controlling for confounding variables like diabetes, hypertension, and depression, people diagnosed with PTSD have two times higher risk of experiencing a myocardial infarction (MI) or stroke [9–14]. Similarly, in a study that evaluated over 8000 veterans between 2005 and 2012, those with PTSD were 47% more likely to develop heart failure (HF) than those without PTSD even after adjusting for age, gender, BMI, diabetes, hypertension, hyperlipidemia, period of military service, and combat service [4]. In addition to hemodynamic changes, previous studies have implicated PTSD-induced impairments of the ANS stimulate a hyperinflammatory state, eventually leading to cardiac dysfunction (Fig. 1). Despite some progress in our understanding of the physiological changes and mechanisms that leave PTSD patients vulnerable to adverse cardiovascular events, our understanding of how PTSD is directly affecting the cardiovascular system is limited.

### Disruption of the Autonomic Nervous System and Inflammatory System Interactions in PTSD and CVD Pathophysiology

Multiple studies have demonstrated dysregulation of the ANS in patients diagnosed with PTSD [15, 16]. The ANS encompasses the sympathetic (SNS) and parasympathetic nervous system (PNS) which are responsible for regulating heart rate, blood pressure, rate of respiration, and body temperature to maintain physiological homeostasis. Understanding interactions between the SNS and PNS and how PTSD associated imbalances may stimulate cardiovascular pathologies is crucial to better understand PTSD-associated CVD.

Disruption of the ANS alone is sufficient to promote adverse cardiac events and CVD, even in the absence of a PTSD diagnosis. For example, in patients diagnosed with depression, ANS dysfunction and inflammatory markers were additive and independent risk factors for long-term CVD mortality [17]. Nonetheless, only a small percentage of the predictive value of depression was attributable to inflammation [17]. Similarly, markers of ANS dysfunction, such as depressed baroreflex sensitivity and heart rate variability (HRV), are strong predictors of cardiac mortality after MI [18].

Angiotensin II (AngII) represents a key mechanistic link between ANS dysfunction, inflammation, and CVD risk, as the renin-angiotensin-aldosterone system (RAAS) [5, 19, 20]. Clinically, antihypertensive therapies such as angiotensin receptor blockers and ACE inhibitors have been associated with reduced PTSD symptom severity, particularly hyperarousal and intrusive symptoms, in predominantly men focused studies [6]. Women typically exhibit greater vagal (parasympathetic) dominance, which is protective against arrhythmias and ischemic injury [7]. However, menopause associated estrogen loss reduces parasympathetic tone and increases cardiovascular vulnerability, resulting in comparable risk between men and women later in life [7]. In a genetic model of renin overexpression and RAAS hyperactivation (Ren2) male rats developed reduced HRV, impaired baroreflex sensitivity, hypertension, myocardial remodeling, and cardiac dysfunction whereas females were relatively protected [21].

Using the repeated social defeat stress (RSDS) model, Case et al. demonstrated a heightened AngII response resulting in elevations in mean arterial blood pressure in wild-type mice compared to Rag2^⁻/⁻^ mice, which lack T-lymphocytes [22]. Interestingly, no differences were observed in pro-social and anxiety-like behavior suggesting immune cell involvement in AngII sensitization [22]. Consistent with these findings, male rats exposed to a resident-intruder paradigm exhibited elevated arterial pressure after AngII infusion that was attenuated by pretreatment with either ACE inhibitor, captopril, or the TNFα synthesis inhibitor, pentoxifylline [23]. These studies further substantiate the combined roles of RAAS signaling and inflammation in stress-induced cardiovascular dysfunction.

Both PTSD and CVD involve inflammatory processes suggesting immune activation may be involved in PTSD-associated cardiovascular pathologies. PTSD contributes to impaired cardiovagal baroreflex sensitivity as well as excessive inflammation as elevated levels of catecholamines stimulate pro-inflammatory cytokine production [24, 25]. Levels of circulating inflammatory cytokines like interleukin (IL)−6, IL-8, IL-17, tumor necrosis factor (TNF)α, and interferon-gamma are elevated in patients diagnosed with PTSD [26–30]. Likewise, PTSD patients have demonstrated elevations in leukocyte and lymphocyte numbers compared to healthy controls [31, 32]. Inhibiting the “fight or flight” arm of the ANS or enhancing parasympathetic tone through vagal nerve stimulation may regulate the immune system and therefore become cardioprotective by preventing chronic and uncontrolled inflammation [33].

Various murine models including the inescapable foot shock (IFS) and RSDS have been used to gain a better understanding of the role chronic inflammation may play in PTSD-associated pathophysiology. Using the IFS fear conditioning model, Sidles et al., demonstrated an increase in circulating inflammatory mediators including IL-6, TNFα, and IFNγ 15-weeks post trauma induction, highlighting a chronic inflammatory environment [34]. Using selective sympathetic denervation of the spleen in the RSDS model, Elkhatib et al. demonstrated circulating T-cell associated cytokines and Th17 polarization and trafficking of splenic monocytes could be diminished despite behavior not being affected [35, 36]. General inflammatory cytokines like IL-6 and TNFα and non-T-lymphocyte immune populations were not affected with splenic denervation implicating the sympathetic system in migration and activation of T-lymphocytes in PTSD [35]. In a RSDS model, acute stress resulted in an increase in Ly6C^hi^ blood monocytes and a decrease in spleen and bone marrow monocytes compared to naïve (control) mice [36, 37]. Interestingly, following a splenectomy, RSDS mice had an increase in circulating monocytes despite a decrease in anxiety symptoms as demonstrated by open field arena (OFA) behavior test [37].

Powell et al., employed a RSDS murine model using male mice and found that splenic CD11c+ dendritic cells (DCs) isolated from male mice exposed to aggressor mice had increased expression of costimulatory molecules including CD44, CD80, and major histocompatibility complex class 1 [38]. When stimulated with lipopolysaccharide (LPS) and CpG DNA, isolated DCs from RSDS mice secreted elevated levels of cytokines compared to controls indicating hyperactivation and possible uncontrolled inflammation [38]. During physiological homeostasis, DCs are susceptible to glucocorticoids leading to decreased survival and antigen presentation [39–42]. As some PTSD patients have blunted glucocorticoid responsiveness, this loss in DC regulation may trigger chronic inflammation after a traumatic event depending on individual glucocorticoid responsiveness [43–45].

While the majority of research investigating the immune system and PTSD pathophysiology does not discuss natural killer (NK) cells in depth, two independent studies of men presenting with PTSD symptomology after the Hanshin-Awaji earthquake in Japan and combat-exposed United States Veterans exhibited similar results with decreased NK cell frequency and activity [46, 47]. In the United States Veterans study, an atypical population of CD56^−^ CD16^+^ NK cells typically found in patients suffering from chronic viral infections was discovered, possibly indicating a senescent response in NK cells after experiencing a traumatic event [47, 48]. Research focused on how neural endocrine signaling intersects with the immune system is needed for researchers and clinicians to better understand the mechanism behind PTSD patient vulnerability to CVD events.

## Sex as a Biological Variable in PTSD and CVD Association

### Sex Differences Based on Clinical Literature

The lifetime prevalence of PTSD has a notable sex disparity: women have a prevalence of approximately 10%, compared to 4% in men [11–13]. Majority of the literature exploring sex differences in PTSD and CVD is clinical and population based and has been disproportionately focused on outcomes in men [16]. Whether cardiac dysfunction, arterial stiffness, and impaired ANS regulation is highly associated in one sex versus the other is not highly reported. While men generally exhibit a higher risk of adverse cardiac events earlier in life, women experience a sharp rise in CVD risk following menopause [49, 50]. Women’s higher prevalence of PTSD, often linked to increased trauma exposure like sexual assault and intimate partner violence, may compound this risk [51]. Some researchers have hypothesized differences in CVD risk between men and women could be due to differences in types of trauma experienced, behavioral symptom presentation, like having increased anxiety-like behaviors prior to experiencing trauma, hormonal differences, and innate ANS function [52, 53]. These differences further demonstrate that sex needs to be highly considered when investigating PTSD and co-morbidities associated with diagnosis.

In a retrospective, longitudinal study of the national Veterans Health Administration electronic medical records, women veterans with PTSD had a 44% increased chance of ischemic heart disease compared to women veterans without PTSD [54]. Similarly, using data of male veterans from the Veteran Affairs Normative Aging Study, Kubzansky et al., found that with increased PTSD severity there was an 18% increased risk of coronary heart disease, even after controlling for depression and risk factors of coronary heart disease [55]. Studies of male dominated populations such as the marines identified a strong association between PTSD and impaired ANS and demonstrated by a reduction in HRV that was further strengthened by a history of prior deployment [56]. Boscarino et al., reported similar results demonstrating men who served in the US Army during the Vietnam War had 20% increased risk for heart disease mortality that increased proportionally with PTSD symptom scoring [57]. All of these studies were further supported by Roy et al., who reported veterans diagnosed with PTSD had an increased risk for developing heart failure (hazard ratio [HR] = 1.47; 95% confidence interval [CI] = 1.13, 1.92) compared to veterans without PTSD even when adjusting for age, diabetes, hyperlipidemia, hypertension, BMI, combat service, and military service period [11]. It is important to note that while women were included the Roy et al. study was composed primarily of men (95%) [11].

Cardiovascular pathology associated with PTSD seems to be associated with hypertension [58–60]. Kibler et al. and Reis et al., both demonstrated that in a population of both men and women, worsening PTSD severity was associated with an increased risk for hypertension [58, 59]. Despite being a mixed population, all of these studies were predominantly men (> 75%). In contrast, Hieda et al., showed that despite not having a medical history of chronic hypertension, cardiovascular, pulmonary, renal, or metabolic diseases, women with PTSD showed signs of diastolic dysfunction demonstrated by an increase in E/e’ values [61]. This effect was not due to age, pregnancy, menopausal status, alcohol, smoking histories, heart rate, or norepinephrine (NE) levels which were comparable between groups. Similarly, Ahmed et al. found young women (18-40 years of age) free from any known CVD but were diagnosed with PTSD had increased central aortic pressure, pulse wave velocity and a decrease in HRV [62]. In a separate study, young women (average 37 years of age) with PTSD demonstrated a decrease in HRV when exposed to either trauma recall or a non-trauma associated stressor (e.g., mental arithmetic) [63]. These findings suggest that in women with PTSD, cardiovascular dysfunction may be independent of increasing afterload and/or catecholamine levels.

In a study published by Ebrahimi et al., women with PTSD experienced ischemic heart disease associated events 2 years earlier than women without PTSD [54]. Similarly, Boscarino et al., reported in a study mainly composed of men (95% of sample size) who served in the Vietnam War, that PTSD diagnosis was associated with an earlier onset of heart disease [57]. In a cohort study of young and middle aged (average age of 30 years) male veterans, Rosman et al. reported that PTSD was associated with earlier onset of atrial fibrillation (AF) after controlling for depression and AF risk factors like age, hypertension, diabetes mellitus, and obstructive sleep apnea [64]. Wiltshire et al. showed young girls (average age of 9 years) repeatedly exposed to violence had lower resting heart rates which were associated with more severe post-traumatic stress symptoms [65]. Within this same study, young boys of the same age did not have a strong association between resting heart rate levels and post-traumatic stress symptoms suggesting traumatic events in prepubescent girls may increase risk of CVD later in life. These studies support the hypothesis that PTSD may stimulate accelerated cardiac aging in both men and women with PTSD, leaving patients vulnerable to experiencing adverse cardiac events earlier in life [9, 57, 64, 66]. While men and women with PTSD are at an increased risk of CVD, the mechanism explaining this vulnerability may be different between sexes. Continued exploration of these differences is critical to improving risk assessment and tailoring interventions for PTSD-associated cardiovascular outcomes in both men and women.

### Sex Differences Based on Animal Models

Using the IFS fear-conditioning model, Corker et al. showed female mice demonstrating a PTSD-like behavioral phenotype exhibited a more fibrotic cardiac phenotype as shown by an enrichment of genes associated with focal adhesion and collagen pathways along with increased myocardial interstitial picrosirius red staining [67]. Interestingly, male PTSD-like mice exhibited an increased trend in cardiac macrophages and matrix metalloproteinase-9 activity 4-weeks post-IFS, indicating a possible role for enhanced macrophage activity in the hearts of male PTSD-like mice. Despite elevations in macrophages, females but not males demonstrated impaired filling and diastolic dysfunction 8-weeks post trauma induction [67]. Using the RSDS model, Cho et al., found greater increases in inflammatory and cardiac tissue remodeling processes in C57BL/6J male mice classified as PTSD-like [68].

Cohen et al. showed immunodeficient male mice experienced more severe PTSD-like symptoms post predator exposure compared to non-immunodeficient mice [69]. When immunodeficient mice were re-supplemented with CD4^+^ CD25^+^ regulatory T-cells, they had a decrease in PTSD-like behavioral symptoms and an improved response to stress further implicating the immune system in PTSD-associated pathology in males [69]. Although not within the context of a PTSD model, a genetic knockout mouse model lacking T-cell receptor β and δ chains and thereby preventing T-lymphocyte activation displayed reduced anxiety-like behaviors compared to mice with intact T-cell function [70]. Specifically, female T-lymphocyte knockout mice spent more time in the illuminated chamber of the light dark box test, while male knockouts spent more time in the center of the open field arena [70]. These findings suggest that while T-lymphocyte depletion can reduce anxiety-like behaviors in both sexes, the behavioral manifestations and sensitivity to anxiety tests may differ between males and females and that the adaptive immune system influences these behaviors. Collectively, these studies indicate that immune modulation, either through T-regulatory cell supplementation or T-cell depletion, may beneficially influence anxiety and PTSD-like symptomology.

Targeted therapies enhancing or minimizing specific immune cell populations may therefore be a better strategy to treat systemic inflammatory effects of PTSD and the behavioral outputs of PTSD diagnosis [70, 71]. Further research is needed to understand the role the adaptive immune system plays with PTSD symptom presentation and downstream physiological effects. Within this research, an emphasis needs to be placed on women/female focused research.

## Current and Experimental Therapeutics for PTSD

The current recommended treatment plan by the American Psychological Association (APA) and Veteran Affairs/Department of Defense (VA/DoD) for people with PTSD includes psychotherapy, selective serotonin reuptake inhibitors (SSRIs), and lifestyle changes [72–74]. More recently, research studies evaluating effects of PTSD have begun to reveal possible mechanisms as to why PTSD patients are vulnerable to pathologies including CVD. These findings have helped researchers and clinicians find ways to treat the somatic effects of PTSD (Table 1).

**Table 1:** Current and experimental therapeutics currently used or in testing for treatment of PTSD

Category	Therapeutic approach	Mechanism/target	Status	Ref
Psychiatric Pharmacology	SSRIs (e.g., sertraline, paroxetine)	Reduces PTSD symptoms; may reduce secondary cardiac risk	FDA approved for PTSD	[75–84]
Pharmacologic - ANS	β-blockers (e.g., propranolol)	Inhibits sympathetic activity; reduces heart rate and BP	Off-label use in PTSD	[85–90]
	Guanethidine	Peripheral sympathetic inhibition	Pre-clinical (murine models)	[37]
	Vagal nerve stimulation	Enhances parasympathetic tone	Ongoing clinical trials	[33, 91]
Pharmacologic - Immune	T-cell phenotype modification	Stress response modulation (adverse outcomes in models)	Pre-clinical (murine models)	[35, 36, 69]
	Monocyte modulation	Blocks immune cell trafficking from spleen	Pre-clinical (murine models)	[37, 71, 92]
	Cytokine production regulation	Decrease cytokine production	Pre-clinical (murine models)	[93, 94]
Lifestyle/ Behavioral	Aerobic exercise	Reduces cardiac stiffness; improves ANS balance	Clinically recommended	[95–97]
	Mindfulness-based stress reduction	Lowers cortisol, improves HRV	Clinically recommended	[98–102]
	Diet modulation, Mediterranean diet	Emphasizes fiber, vitamins, healthy fats over processed foods	Clinically recommended	[103–106]

*ANS* autonomic nervous system, *BP* blood pressure, *HRV* heart rate variability, *PTSD* post-traumatic stress disorder, *SSRI* selective serotonin reuptake inhibitor

### Selective Serotonin Reuptake Inhibitors

The only Federal Drug Administration (FDA) approved treatment for PTSD are SSRIs; sertraline (Zoloft) and paroxetine (Paxil) [75, 76]. SSRIs inhibit the uptake of serotonin, resulting in an elevation of total serotonin levels and minimizing depression and anxiety symptoms [74]. While beneficial behavioral outcomes have been demonstrated with sertraline and paroxetine, clinical literature is inconclusive on the effects of SSRIs on inflammation and CVD outcomes [77–83, 107–109]. One reason for the inconclusive outcomes could be data regarding effect of SSRIs on the heart is dependent on what context SSRI and cardiac health are being investigated [110]. The current literature is limited when comparing the effects of SSRI on cardiac health in men versus women as much of the focus has been on the use of SSRIs in the context of pregnancy and in post-partum women. For example, while paroxetine has been shown to be safe for use in post-MI patients [111, 112], according to the American College of Obstetricians and Gynecologists, paroxetine should not be prescribed to expectant mothers for prenatal depression to avoid potential heart defects in the developing fetus [63]. Zhu et al., further demonstrated that paroxetine stunted zebrafish larvae growth, decreased cardiac output, and increased cardiac inflammatory gene expression when zebrafish were exposed to paroxetine *in utero* [64]. Sun et al. showed paroxetine treatment resulted in a partial decrease in cardiac hypertrophy in patients with hypertension [113]. The authors proposed that this decrease in hypertrophy may be due to increases in the sensitivity of adrenergic receptor beta 1 (*Adrb1*) and by blocking G protein-coupled receptor kinase 2 (*Grk2*) mediated Adrb1 activation [113]. Male rats treated with paroxetine after MI had decreased cardiac fibrosis, Grk2 and Tgfβ expression, inflammatory proteins, and cardiac macrophages [54]. Survival increased and left ventricular morphology and function remained stable in Wister male rats exposed to doxorubicin (DOX) in combination with paroxetine, demonstrating a potential protective-mechanism for paroxetine against DOX-induced organ toxicity [114]. In all these studies indicate a potential protection against cardiac hypertrophy and fibrosis with paroxetine in adults that may be detrimental during development.

Sertraline has no known risks associated with cardiovascular conditions and has been considered safe to use in patients with HF suffering from anxiety and depression [115–118]. Sertraline is also commonly prescribed to pregnant women suffering from prenatal depression [84]. Despite this, Lu et al. revealed male C57BL/6J mice exposed to sertraline *in utero* and 14 days after birth presented with decreased heart rate whereas female mice had decreased ejection fraction [84]. Multiple studies have repeated the claims Lu et al. reported stating that sertraline can affect fetal and newborn cardiac health [119–122]. In opposition to these studies, Kolding et al. found no significant differences in fetal cardiac function when women were prescribed sertraline in gestational week 25 and 26 [123]. The findings of these studies raise questions regarding the use of sertraline to control symptoms of prenatal depression and whether there is a risk for adverse cardiac events in children if exposed *in utero*. Inconclusive results between animal and clinical studies highlight the need to better understand the effect of SSRIs on the heart particularly when considering effects on development.

### Neurologic Interventions

Interventions that target the ANS are currently under investigation at the preclinical stage [124, 125]. Vagal nerve stimulation is currently being evaluated in patient populations for the possible direct effect on behavior outcomes in PTSD patients. A study published recently in Brain Stimulation from Powers et al., showed that PTSD behavioral symptoms clinically improved and this improvement persisted 6 months after vagal nerve stimulation treatment [91]. Majority of the participants were younger women (8/9 participants; aged 34–63 years) [91]. Surprisingly, vagal nerve stimulation therapy led to no participants meeting the criteria for PTSD diagnosis 6 months after therapy was received [91]. While vagal nerve stimulation benefits PTSD behavior symptomology, it has also been shown to reduce pro-inflammatory cytokines like IL-6 in patients with PTSD [126]. Majority of the literature investigating vagal nerve stimulation treatment on inflammation is within inflammatory bowel disease and epilepsy, where inflammatory cell populations and cytokines decrease after vagal nerve stimulation [127–131]. Vagal nerve stimulation also increases parasympathetic signaling, therefore increasing HRV potentially providing beneficial cardiovascular effects [33, 132–134]. In opposition, some studies have shown a decrease in HRV with vagal nerve stimulation [135, 136]. These studies have been conducted in patients suffering from epilepsy and immediately following autonomic challenges like caloric intake.

Propranolol is a first-generation beta blocker that is traditionally used to reduce blood pressure and heart rate through inhibition of SNS [137]. While FDA approved, propranolol is classified as off label use for PTSD. Some report propranolol reduces symptom severity only when administered prior to a traumatic event while others report beneficial effects for reducing behavioral symptom severity after PTSD diagnosis [85–90]. This could be due to the physiological effect propranolol has, like regulating heart rate. Propranolol blocks beta-adrenergic receptors, reducing SNS activity and lowering heart rate, blood pressure, and physiological arousal associated with PTSD [138]. By dampening sympathetic dominance, propranolol may enhance PNS activity and HRV, which is linked to better emotional regulation and stress resilience [138, 139]. Guanethidine, a peripheral sympathetic inhibitor, has been tested in PTSD murine models to evaluate effects on behavior and immune modulation [37]. McKim et al. demonstrated that guanethidine may inhibit the recruitment of monocytes and blunt anxiety-like behaviors in mice, preventing damage to the heart and other organs [37]. No studies to date have evaluated the effect of guanethidine on CVD outcomes in the setting of PTSD. Further research is needed to validate these claims in larger and more diverse sample sizes. Neuromodulation therapeutic trials in PTSD rarely stratify outcomes by sex, leaving critical gaps in our understanding on whether treatments produce differential responses between sexes.

### Immune System Modulation

Immune modulating treatments represent a promising alternative to traditional CVD treatments. In the setting of PTSD, the majority of studies have investigated the potential of immunomodulators on behavior and physiology in pre-clinical models. Nonetheless, studies have demonstrated promise in potentially revolutionizing the management of PTSD-induced cardiovascular events [35, 37, 93, 94]. Recent studies have suggested cyclooxygenase-2 (COX-2) inhibition could reduce a variety of stress-induced behavioral pathologies potentially through negative regulation of proinflammatory cytokine production [93, 94]. By using a method of splenic denervation that effectively reduced splenic tyrosine hydroxylase and NE, Elkhatib et al. demonstrated sympathetic nerves regulate stress-induced splenic T lymphocyte inflammation but play less of a role in the behavioral and non T lymphocyte inflammatory phenotypes [35]. Similarly, administration of guanethidine, a peripheral sympathetic inhibitor that prevents the release of NE, decreased monocyte recruitment in circulation and to stress regions of the brain further solidifying a link between PTSD and the SNS [37]. Elevated numbers of classical CD14^+ +^ CD16^−^ monocytes have been shown to predict future cardiovascular risk independent of other risk factors [92], suggesting SNS mediated decrease in monocytes may be cardioprotective.

With PTSD there is a robust activation of the immune system and inflammatory mediators some of which have been shown to be sex dependent [140–142]. While multiple studies have included women, only a few have probed for sex differences in inflammatory status in comparison to age- and sex-matched controls. Although Kuffer et al., showed no difference in overall IL-6 or TNFα levels in PTSD patients versus controls, overnight trajectories of cytokine levels did differ though no sex differences were observed [141]. In contrast, Lalonde et al., found men had higher concentrations of pro-inflammatory cytokines including IL-6, IL-1𝛽, TNF, and IFNγ [140]. Interestingly, in a study that only evaluated PTSD women after sexual assault, no difference were observed in inflammatory markers however, after 1 year, there was a significant increase in IL-1β, monocyte chemoattractant protein-1, TNF-α, and c-reactive protein in PTSD women highlighting the impact that time may have on the chronic inflammatory status of PTSD [142]. While these studies highlight potential sex differences in inflammatory status, the absence of evidence stratified by sex in immunomodulatory and anti-inflammatory treatment prevents the development of biologically relevant interventions. Future studies are needed to determine if immune specific modulators could protect stress-induced CVD in both sexes.

### Lifestyle Changes

In addition to treatments such as SSRIs, clinicians strongly recommend incorporating targeted lifestyle changes into the care plan for patients with PTSD [143, 144]. Aerobic exercise, for instance, not only alleviates common behavioral symptoms like anxiety, insomnia, and hyperarousal, but also improves cardiovascular health by reducing cardiac stiffness and supporting ANS balance [95–97]. Similarly, practicing mindfulness-based stress-reduction techniques such as spending time in nature, engaging in breathing exercises, and meditating can help regulate cortisol levels and enhance HRV [98–102]. A common lifestyle intervention suggested for PTSD patients are alterations in diet [104]. Van den Berk-Clark et al. discovered using a meta-analysis of over 19 studies and 1.6 million participants that veterans and civilians with a PTSD diagnosis were more likely to be obese and eat fast food [103]. While results are mixed, some studies suggest that gut microbiota may have a link to PTSD severity and cardiovascular health [105–145]. Highly processed foods often lack the nutrients and fiber intake needed to maintain a healthy gut microbiota, leading to chronic low-grade inflammation [146]. Whether or not improving gut microbiota would protect PTSD patients from CVD risk has not been studied in full.

These lifestyle interventions offer meaningful behavioral and cardiovascular benefits, making them a valuable addition to traditional therapies. Implementing these changes empowers patients and their support systems to take an active role in improving overall health and well-being alongside standard treatment.

## Conclusions

The association between PTSD and CVD has been well characterized throughout the clinical landscape but mechanistic answers as to why this association is so prevalent is understudied. Gaining a better understanding behind the association of PTSD and CVD would improve our knowledge on how current FDA approved medications including SSRIs may affect cardiovascular health and aid in identifying novel treatment strategies.

## Data Availability

Data availability is not applicable to this article because no new data were created or analyzed in this study.

## Abbreviations

PTSD: Post traumatic stress disorder
CVD: Cardiovascular disease
BMI: Body mass index
AF: Atrial fibrillation
IL-: Interleukin-
ANS: Autonomic nervous system
HF: Heart failure
MI: Myocardial infarction
IFS: Inescapable foot shock
TNFα: Tumor necrosis factor-alpha
RSDS: Repeated social defeat stress
LPS: Lipopolysaccharide
DCs: Dendritic cells
NK: Natural Killer cells
HRV: Heart rate variability
OFA: Open field arena
PNS: Parasympathetic
SNS: Sympathetic nervous system
AngII: Angiotensin II
RAAS: renin-angiotensin-aldosterone system
ACE-I: ACE inhibitor
LV: Left ventricle
SSRIs: Selective serotonin reuptake inhibitors
APA: American Psychological Association
VA/DoD: Veteran Affairs/Department of Defense
FDA: Federal Drug Administration
NSAIDs: Non-steroidal anti-inflammatory drugs
GRK2: G protein-coupled receptor kinase 2
ADRB1: Adrenergic receptor beta 1
DOX: Doxorubicin
COX-2: Cyclooxygenase-2
NE: Norepinephrine
